# Beliefs, knowledge and the impact of COVID19 on menopause therapies in Spanish women: COMEM-treatment study

**DOI:** 10.1186/s12905-020-01151-x

**Published:** 2020-12-28

**Authors:** Laura Baquedano, Andrea Espiau, María Fasero, Silvia Ortega, Isabel Ramirez, Nicolás Mendoza, The COMEM Study Spanish investigators, Laura Baquedano, Andrea Espiau, Silvia Ortega, Leyre Ruiz, Marta Lamarca, Yasmina José, Patricia Rubio, Francisco Villalobos, Amparo Borque, Peña Dieste, Lourdes Gabasa, Virginia Roy, María J. Puente, Marta Chóliz, Laura Cotaina, Isabel Negredo, Pilar del Tiempo, Hortensia Yagüe, Mónica Hernnández, Pilar D. Tajada, María Fasero, I. Ramirez, Luisa Gutiérrez, Fernando Colmenarejo, Pluvio J. Coronado, Teresa Aznar, Jesús Presa, Placido Llaneza, Rafael Sánchez-Borrego, Santiago Palacios, Ana R. Jurado

**Affiliations:** 1 Gynecology Department of Miguel, Servet University Hospital, Paseo Isabel La Católica 1-3, 50009 Zaragoza, Spain; 2 Service of Obstetrics and Gynecology, Hospital Sanitas La Zarzuela, Madrid, Spain; 3 Sexual and Reproductive Health Service, UGC Dr Cayetano Roldan San Fernando Health Centre, Cadiz, Spain; 4 Department of Obstetrics and Gynecology, University of Granada, Granada, Spain

**Keywords:** Menopause hormone therapy, Knowledge, Menopause, COVID-19, Confinement

## Abstract

**Objective:**

To study what women think about menopause treatments and assess their knowledge about them. To analyze adherence to treatment during COVID-19 confinement as a secondary objective.

**Methods:**

A multi-center cross-sectional observational study was conducted using a survey of 2500 women between January and June 2019. This was administered following a non-probability sampling procedure including women between 35 and 75 years. An extension study was conducted during the coronavirus pandemic, between March and June 2020.

**Results:**

The responses of 2355 surveyed women were analyzed. Of this sample, 42% knew about menopause hormone therapy (MHT). The most frequently identified indication was the treatment of hot flashes (65.6%). The MHT risks most frequently perceived were weight gain (24.2%) and breast cancer (21.7%); the main reason for rejecting MHT was a lack of information (96.1%). Comparative analyses were conducted according to age, menopausal status, type of menopause, place of residence, type of health care and level of education. During the coronavirus confinement period, 85 women using MHT were located, of which 84.7% continued it.

**Conclusions:**

Women hold certain false beliefs about menopause, and their knowledge of the available treatments is somewhat limited. Adherence to MHT during the COVID-19 confinement in Spain has been high.

## Background

Menopause is a period of the life cycle marked by several modifications, among the most notable of which are certain biological events that are influenced by hormonal, social, family and personal alterations, all of which can lead to adaptive difficulties in some women [1].

Accurate information about everything that occurs and is related to this stage will contribute towards normalizing the menopause as just another phase within the woman's life cycle, and, moreover, it will help to identify women who need medical attention due to the presentation of symptoms that affect their quality of life [2]. As part of this information, acquiring informed knowledge of the most important clinical aspects, of their possible consequences, as well as the available therapeutic alternatives are basic pillars for ensuring the autonomy of women so that their decisions can be made without the influence of stereotypes and myths [3].

There is limited data about women's attitudes and experiences around menopause in Spain, and those that do exist are very incomplete and only related to the fear that menopause hormone therapy (MHT) has generated among the population [4–6]. The primary objective of this study was to assess beliefs and knowledge among women in Spain in this regard. Therefore, we designed a study based on a survey named the COMEM study (“COnocimiento de las Mujeres Españolas en Menopausia”, in its Spanish acronym). This questionnaire was developed for this study *(Supplementary material)*. In addition, as a secondary objective, we carried out an extension of the study to evaluate the adherence of women to MHT during the period of confinement imposed due to the coronavirus (COVID-19) pandemic.

## Methods

A multi-center cross-sectional observational approach was adopted to analyze the findings of an anonymous survey administered to 2500 women between January and June 2019. The extension study was carried out during the coronavirus pandemic, between March and June 2020 (during the state of alarm in Spain).

### Survey design

The questions included in the survey were chosen by consensus among a team of seven menopause experts. The complete questionnaire consisted of 22 questions written in a closed format, in simple and clear language to facilitate the women’s response. Only one open-ended question was asked about hobbies and leisure time. The questionnaire was structured into three clinical domains: clinical-biological aspects of menopause and associated general health risks (COMEM-clinical study); therapies in menopause, (COMEM-treatment study); and sexuality and Menopause Genitourinary Syndrome (COMEM-MGS study).

The questionnaire was administered following a non-probability or convenience sampling procedure. Specifically, the survey was offered to women aged between 35 and 75 years that attended the gynecology consulting-rooms of the ten participating centers. The selection of patients was random and systematic; upon arrival at the clinic, women were selected and agreed (or not) to participate in the study. Women who suffered from any kind of physical, psychological or language incapacity that would hinder their understanding of the survey questions were excluded from the study. The questionnaire was pre-tested among 20 women to obtain feedback on the appropriateness, sequencing, and understanding of the questions.

Following the declared state of emergency in Spain due to the coronavirus pandemic (COVID-19), women experienced a period of prolonged confinement. As a complement to the proposed study, women with MHT who had completed the survey were asked about their adherence and attitude to treatment during this period. For this purpose, they were contacted by telephone.

The research was conducted in accordance with Good Clinical Practice standards and the current revision of the Declaration of Helsinki.

The study was approved by the Research Ethics Committees of Aragon, Spain (C.I. PI18/374) and confirmed by each participating Center and was included in the Spanish Menopause Society National Research Network (REIM).

### Statistical analysis

Quantitative variables are presented using means and standard deviations, whilst categorical variables are reported in frequencies and percentages. For each response to the questionnaire, a statistical inference was made using Chi-Square test/Fisher's exact test or Student’s T-test. The following factors were taken into account as they were considered possible confounding variables: age, place of residence (> or < 100.000 inhabitants), menopause status (pre/postmenopausal), type of menopause (natural/surgical), level of education (primary-secondary/university) and type of medical care (public/ private). Postmenopausal status was defined as the absence of menses for over 12 consecutive months. To assess the association between the responses and the variable "age", a linear regression analysis was conducted to reveal the magnitude of the responses per year of age. The level of statistical significance was set at *p* < 0.05.

The data were collected and codified using IBM Statistics Process Social Sciences 22.0 for Windows (Copyright© SPSS Inc., 2013) for subsequent statistical analysis.

## Results

Anonymous surveys were handed out to 2500 women who agreed to participate voluntarily once the objective of the study had been explained. Of these, 145 surveys were returned blank, so they were excluded from the final analysis, which included data from 2355 completed surveys.

The socio-demographic characteristics of the 2355 surveyed women are shown in Table 1. Of the sample, 89.2% were from public health centers and the remainder (10.8%) were receiving private health care. The present study shows the results obtained from the COMEM-treatment study.

**Table 1:** Socio-demographic characteristics of the sample

	n	%
Age		
Up to 45 years	621	26.4
From 46 to 55 years	818	34.7
From 56 to 65 years	661	28.1
Over 65 years	236	10.0
Health center		
Public assistance	2101	89.2
Private assistance	254	10.8
Menopausal status		
No	973	41.3
Yes	1362	57.8
Do not know/do not answer	20	0.8
Early menopause		
Yes	41	1.7
No	1142	48.5
Do not know/do not answer	1172	49.7
Type of menopause		
Natural	1127	47.9
Surgical	168	7.1
Do not know/do not answer	1060	45
Level of study		
Basic/medium	1156	49.1
University	864	36
Do not know/do not answer	13	0.6
Place of residence		
< = 100.000 inhabitants	633	26.9
> 100.000 inhabitants	1443	61.3
Do not know/do not answer	279	11.8

The responses to the items of the questionnaire are shown in Table 2. 82.8% of the surveyed women showed interest in the subject of menopause. Most of the women identified physical exercise (66.8%) and a diet rich in calcium and vitamin D (63%) as beneficial therapeutic alternatives for the management of menopause, 5.4% had no knowledge of any menopause treatment, and 5.1% believed that there are no treatments available for the relief of menopause symptoms.

**Table 2:** Menopause treatment beliefs and knowledge survey

	n	%
Are you interested in menopause?		
Yes	1949	82.8
No	326	13.8
DK*	80	3.4
What treatments do you know to treat the symptoms of menopause?		
No treatment exists	119	5.1
Vaginal lubricants	1156	49.1
Hormonal treatment	1257	53.4
Antidepressants	347	14.7
Phytotherapy: soyisoflavones, red clover	669	28.4
Acupuncture	149	6.3
Exercise: walking, yoga, etc.	1573	66.8
Food rich in calcium and vitamin D	1484	63.0
DK*	126	5.4
Do you know what menopause hormone therapy is?		
Yes	994	42.2
No	1251	53.1
DK*	110	4.7
What are the indications for menopausal hormone therapy?		
Delay ageing	229	9.7
Improve hot flushes	1544	65.6
Improve your sleep	740	31.4
Bones and joints improvement	1161	49.3
Improve dyspareunia	317	13.5
Treating Depression	312	13.2
DK*	577	24.5
Would you use menopausal hormone therapy if your gynecologist advised you to do it?		
Yes	1664	70.7
No	535	22.7
DK*	156	6.6
If the answer is no, specify the reasons		
Fear and distrust	163	30.5
No need to treat it	89	16.6
I would need more information about it	514	96.1
Economic resources	24	4.4
DK*	26	4.8
Do you know what the risks of hormone therapy are?		
It doesn't have any	319	13.5
Breast cancer	510	21.7
Risk of thrombosis	444	18.9
Osteoporosis	166	7.0
Weight gain	569	24.2
Cancer of the uterus	273	11.6
DK*	964	40.9
Your sources of information on menopause are		
Friends	963	40.9
Magazines and press	593	25.2
Healthcare professionals	1167	49.6
Family members	799	33.9
Television	349	14.8
Internet	688	29.2
DK*	145	6.2
If you have marked healthcare professionals, please specify among these		
Gynecologist	793	67.90
Midwife	335	28.70
Nurse	264	22.60
Family doctor	404	34.60
DK*	23	1.90
If you had any questions about menopause, who would you go to?		
Primary health care	639	27.1
Specialty care	1547	65.7
DK*	169	7.2
Specify the reason		
Easy access	806	34.2
Trust	442	18.8
Knowledge in menopause	814	34.6
Others	52	2.2
DK*	241	10.2

Almost half of the surveyed women (42%) were aware of MHT. The most frequently identified indication for MHT was treatment for hot flashes (65.6%) followed by bone and joint strengthening (49.3%) and sleep improvement (31.4%). Improved sexuality or mood was identified in 13.5% and 13.2% of the participants respectively. One in four surveyed women (24.5%) were unaware of any of the indications for MHT.

The MHT-associated risks most frequently perceived by women were weight gain (24.2%) followed by breast cancer (21.7%). The risk of thrombosis associated with MHT was identified in only 18.9%, particularly in the case of those with a higher education level; whilst 40.9% did not know of any risks associated with MHT.

With regard to the source of information, 50.4% used non-professional sources, and in case of needing to clarify any doubts concerning menopause, most women chose the gynecologist or the midwife (67.9%), whilst 70.7% stated that they would use MHT if advised by their gynecologist. Of the remainder, 96.1% said that they would not do so due to lack of information, with fear or distrust being the second most frequently reported reason for refusing MHT.

Comparative analyses were conducted according to the age of each woman at the time of the survey, her menopausal status, the type of menopause, her place of residence, the type of health care she received, and her level of education. Statistically significant differences were found regarding these variables for several of the given responses (Additional Files 1 and 2: Tables 3, 4). For this comparative analysis, missing or unanswered responses were eliminated.

During the coronavirus confinement period, we were able to access 85 women using MTH; none of whom were infected with COVID-19. Indications for treatment had been confirmed by a gynecologist in all cases. Of this sample, 38 (44.7%) were using combined estrogen and progestogen treatment pills, 33 (38.8%) were using estradiol spray with natural progesterone and 14 (16.5%) were using estradiol spray as monotherapy. Finally, 84.7% (n = 72) of these women adequately followed their treatment during the confinement period. There were 9 women who suspended MHT due to supply problems (all with spray estradiol) and 4 women due to the fear of leaving home to buy it. All of the women considered it important to take MHT and to not stop doing so during the period of confinement.

## Discussion

The main finding of this survey is that the women in our analyzed sample have poor knowledge and false beliefs about treatments in menopause, essentially MHT. Thus, there is considerable potential for improvement in this regard. High educational level, use of private health care, postmenopausal status, and older age were factors associated with increased knowledge of MHT. To date, this is the largest survey-based study to evaluate the beliefs and knowledge about menopause and its treatments in Spain. This study is also the first to analyze the attitudes held by women towards MHT during the state of alarm declared in response to the coronavirus pandemic.

The proportion of women in our survey who knew about MHT (42%) is lower than that reported in other studies with similar populations, where the estimated proportion of women who did know about its existence reached 70–82% [8–10]. The study conducted in Belgium was based on an online survey of 600 postmenopausal women and almost all participants (82%) knew about the various treatment options, including MTH. In the North American Menopause Society survey, the percentage of women that have some knowledge of MTH reached 84%[10]. In contrast, other studies such as the one conducted with a Brazilian cohort or a study carried out in Central America showed that 60.2% and 72.8% (respectively) of the surveyed women were unaware of the usefulness of MHT [11, 12]. This implies that there are important cultural and geographical differences regarding knowledge of MHT.

It is noteworthy that only 10% of the women in our study pointed to improvements in sexuality as a possible indication for MHT, an issue that had not been explored in other similar studies [7–12]. However, there are many women with sexual dysfunction associated with menopause, primarily lack of desire and dyspareunia, for whom MHT may be beneficial [13–16]. A study of menopausal women in our country showed that women with better knowledge of menopause also had better indicators of sexual health [17]. The specific questions asked in the survey about menopause sexuality and genitourinary syndrome of menopause, will be exposed in another study.

It is also interesting to note that nearly one-third of the respondents stated that they would not use MHT in spite of medical advice, and most of them would not do so because of a lack of information. Almost all of the similar reviewed studies agree that when more information is available to women, this generates greater demand for and use of MHT [3, 18–22]. Nonetheless, women using MHT consider it important for improving their quality of life, and even during the COVID-19 confinement period, most women using MTH continued with their treatment. Stress and forced lifestyle changes during the confinement could, in menopausal women, lead to an aggravation of symptoms. To our knowledge, this is the first study to evaluate the opinion of women using MHT and their adherence to treatment during the coronavirus pandemic. Other specific populations of women, such as pregnant women or women with osteoporosis, have been studied during the COVID-19 confinement period. A decrease in quality of life has been observed in these groups of women during this period. The authors emphasize the need to implement specific training education and health care programs [23, 24].

The risks of MHT that were most frequently identified among the surveyed women were weight gain and breast cancer. Menopausal status, older age, use of private health care, place of residence (above 100.000 inhabitants), and high educational level were associated with a stronger likelihood of identifying breast cancer as a risk associated with MHT, whilst use of public health care and lower educational level were associated with weight gain as an identified risk factor. In the literature, the adverse outcome most frequently associated with MHT is breast cancer, particularly in studies conducted in the years following the publication of the WHI trial [9, 11, 25, 26]. Weight gain is not usually included as an option in closed-response menopausal knowledge surveys, which could explain why this has not been reported in the literature thus far. However, this is not a surprising finding, since this is a common reason for gynecological consultation, as in the case of other hormonal treatments such as contraceptives. On the other hand, the risk of thrombosis associated with MHT was identified by a low percentage of women (18.9%), and these women generally had a higher educational level. These results indicate that myths still persist in the studied sample and there is a lack of important information about the real risks of MHT, a deficit that appears to have been particularly evident during the COVID 19 pandemic [27].

The use of phytotherapy in menopause was unknown to most of the surveyed women. However, most of the women identified exercise and a diet rich in calcium and vitamin D as therapeutic options. Postmenopausal status, older age and high cultural level were associated with a greater likelihood of knowing the health benefits of a healthy lifestyle. Indeed, the culture and lifestyle habits of each country could have an impact on women's beliefs and the importance assigned to such habits when it comes to experiencing a healthy menopause [10, 28, 29]. Perhaps the strongest proponents of the link between lifestyle factors and menopause are Asian women. In an epidemiological survey conducted in 2015, more than 90% of a sample of these women believed that their menopausal symptoms could be effectively treated by lifestyle modifications such as diet and exercise or by natural treatments [30].

One limitation of our study could be the possible selection bias. In order to minimize this, the sample size was increased and the opportunity to participate was given to both women who attended the centers for a consultation of their own (whether menopause-related or not) and women who were accompanying others (provided that they met the inclusion criteria). Nonetheless, it is assumed that the conclusions cannot be extrapolated to the general population but are instead specific to the sample included in the study (although this might be useful as an exploratory study of the general population). Another possible limitation of our study is the choice of questionnaire. There is no questionnaire in the Spanish language that has been sufficiently recognized and validated to measure women's knowledge of the menopause. In Spain, Padilla et al. (2000) designed a questionnaire with 56 items to assess the knowledge of women in menopause, but its use has not been widespread, and given the time elapsed and the multiple changes that have occurred since then, it is no longer used.

It has been reported that the benefits of treatment are transmitted primarily by health professionals, whilst the risks are communicated through the media [5]. Previous studies carried out in our country have pointed out that the majority of sources of information on menopause are friends, family or the media, while only a small percentage (15–25%) used medical sources of information [5–7]. The data from our study point in another direction, since nearly half of the women reported seeking information from health professionals (preferably specialists in gynecology), particularly those women with a higher level of education, of older age or within menopause. These findings seem encouraging and confirm the finding of a previous study conducted in our country, which, in addition, found that a more positive attitude towards menopause was observed when the woman had received the information from a gynecologist [4].

During the COVID-19 pandemic, the importance of the work carried out by health professionals—and the attendant implications for public health—has become evident. This has probably led to an improvement in the doctor-patient relationship and an increase in confidence in clinicians [31]. Thus, now more than ever, there is a great opportunity to change certain beliefs that are held by women and to improve their knowledge of MHT. Before this treatment is prescribed, it is important to assess their level of knowledge and their attitudes. Moreover, providing women with the correct information needed to understand the reasons why the treatment is indicated, reinforcing its benefits, clarifying possible risks, and resolving doubts, will be key to helping women to lose unjustified fears of MHT and freely choose the best option to improve their quality of life. In public centers, for instance, it could be of interest to develop educational materials aimed at younger, premenopausal women with lower levels of education, since these appear to represent the most vulnerable groups.

## Conclusions

Although women show an interest in the issue of menopause, they hold certain false beliefs, and their knowledge about treatments is somewhat limited. The demand for and use of treatments such as MHT depends largely on the acquisition of this knowledge. Consequently, accurate information, including a balanced view of the risks and benefits, is essential if women are to make informed decisions about treatment. This information should be provided by healthcare professionals, with the gynecologist being identified as the reference on menopause issues in this survey. Adherence to MHT during the coronavirus confinement period in Spain has been high, because patients receiving this treatment consider this to be important for their well-being and quality of life.

## Supplementary information

**Additional file 1.** Table3. Comparative analyses according to the type ofhealth care, place of residence, and level of education.

**Additional file 2.** Table 4. Comparativeanalyses according to menopausal status, age and type of menopause.

## Data Availability

The datasets during and/or analysed during the current study available from the corresponding author on reasonable request.

## Abbreviations

MHT: Menopause hormone therapy
COMEM: “COnocimiento de las Mujeres Españolas en Menopausia”, in its Spanish acronym
