# Supplementary material for: Beliefs, knowledge and the impact of COVID19 on menopause therapies in Spanish women: COMEM-treatment study
Source: BMC Womens Health. 2020 Dec 28;20:277. doi: 10.1186/s12905-020-01151-x (PMC7768270; doi:10.1186/s12905-020-01151-x)
Supplement: Supplementary file 1 — Additional file 1. Table3. Comparative analyses according to the type ofhealth care, place of residence, and level of education. [file 12905_2020_1151_MOESM1_ESM.docx]

Table 3. Comparative analyses according to the type of health care, place of residence, and level of education

|  |  | Health care type %(n*) | | | | Place of residence %(n*) | | | | Level of study %(n*) | | | |
| --- | --- | --- | --- | --- | --- | --- | --- | --- | --- | --- | --- | --- | --- |
|  |  | Public | Private | OR (IC 95%) | p value | < 100.000 population | > 100.000 population | OR | p-value | Academic | Basic//medium | OR (IC 95%) | p-value |
| Are you interested in menopause? | Yes | 85,8%(1739) | 84,7%(210) |  | 0,631 | 89,5%(553) | 85,7%(1201) | 1,424 (1,058 - 1,917) | 0,019 | 86,6%(735) | 85,2%(1204) |  | 0,369 |
| What treatments do you know to treat the symptomsof menopause? | |  |  |  |  |  |  |  |  |  |  |  |  |
| No treatment exists | | 5,5%(110) | 3,7%(9) |  | 0,289 | 3,3%(20) | 5,3%(73) | 0,603 (0,364 - 0,998) | 0,050 | 6,8%(24) | 2,9%(94) | 0,526 (0,366-0,755) | <0,001 |
| Vaginal lubricants | | 51,1%(1014) | 58,4%(142) | 0,742 (0,567 - 0,972) | 0,035 | 51,2%(313) | 54,1%(744) |  | 0,242 | 47,7%(493) | 58,9%(659) | 1,329 (1,191-1,483) | <0,001 |
| Hormonal treatment | | 55,4%(1101) | 64,2%(156) | 0,694 (0,526 - 0,915) | 0,009 | 55,5%(339) | 58,1%(798) |  | 0,302 | 49,2%(572) | 68,3%(681) | 1,666 (1,479-1.4876) | <0,001 |
| Antidepressants | | 16,0%(317) | 12,3%(30) |  | 0,160 | 17,2%(105) | 15,1%(208) |  | 0,257 | 15,4%(132) | 15,8%(213) |  | 0,816 |
| Phytotherapy: soyisoflavones, red clover | | 30,1%(597) | 29,6%(72) |  | 0,941 | 32,1%(196) | 30,7%(422) |  | 0,564 | 25,5%(314) | 37,5%(353) | 1,398 (1,257-1,555 | <0,001 |
| Acupuncture | | 6,7%(133) | 6,6%(16) |  | 1,000 | 8,5%(52) | 6,3%(86) |  | 0,070 | 6.5%(58) | 6,9%(90) |  | 0,699 |
| Exercise: walking, yoga, etc. | | 70,4%(1398) | 72,0%(175) |  | 0,655 | 72,5%(443) | 71,7%(985) |  | 0,745 | 67,5%(632) | 75,5%(935) | 1,285 (1,130-1,461) | <0,001 |
| Food rich in calcium and vitamin D | | 66,6%(1322) | 66,7%(162) |  | 1,000 | 66,3%(405) | 68,5%(941) |  | 0,349 | 64,4%(588) | 70,3%(890) | 1,186 (1,052-1,335) | 0,004 |
| Do you know what menopause hormone therapy (MHT) is? | Yes | 43,4%(869) | 51,2%(125) | 0,731 (0,560 - 0,954) | 0,024 | 43,3%(261) | 47,5%(661) |  | 0,087 | 56,8%(476) | 36,7% | 1,655 (1,485-1,844) | <0,001 |
| What are its indications? | | | | | |  |  |  |  |  | |  |  |
| Delay ageing | | 12,8%(203) | 13,5%(26) |  | 0,820 | 12,2%(57) | 13,0%(145) |  | 0,681 | 12,6%(90) | 12,6%(137) |  | 0,831 |
| Improve hot flushes | | 86,4%(1370) | 90,2%(174) |  | 0,176 | 86,3%(402) | 87,4%(972) |  | 0,565 | 90,0%(641) | 90,0%(895) | 1,358 (1,109-1,662) | <0,001 |
| Improve your sleep | | 40,8%(646) | 48,7%(94) | 0,725 (0,537 - 0,978) | 0,037 | 41,6%(194) | 42,6%(474) |  | 0,738 | 44,0%(313) | 44,0%(421) |  | 0,090 |
| Bones and joints improvement | | 65,2%(1034) | 65,8%(127) |  | 0,936 | 63,7%(297) | 67,4%(750) |  | 0,161 | 64,2%(457) | 64,2%(698) |  | 0,392 |
| Improve dyspareunia | | 17,4%(276) | 21,2%(41) |  | 0,196 | 21,7%(101) | 16,5%(183) | 1,405 (1,071 - 1,843) | 0,015 | 16,4%(117) | 16,4%(197) |  | 0,227 |
| Treating Depression | | 17,7%(280) | 16,6%(32) |  | 0,764 | 21,2%(99) | 16,7%(186) | 1,343 (1,023 - 1,763) | 0,037 | 15,9%(113) | 15,9%(196) |  | 0,142 |
| Would you use MHT if your gynecologist advised you | Yes | 75,5%(1486) | 77,4%(178) |  | 0,570 | 77,6%(450) | 74,6%(1021) |  | 74,3% | 76,7%(615) | 76,7%(1040) |  | 0,222 |
| If the answer is no, specify the reasons: | |  |  |  |  |  |  |  |  |  |  |  |  |
| Fear and distrust | | 29,3%(140) | 45,1%(23) | 0,504 (0,281 - 0,906) | 0,025 | 32,5%(41) | 28,8%(99) |  | 0,428 | 34,5%(71) | 34,5%(91) |  | 0,157 |
| No need to treat it | | 16,7%(80) | 17,6%(9) |  | 0,845 | 18,4%(23) | 16,5%(57) |  | 0,677 | 15,9%(33) | 15,9%(53) |  | 0,784 |
| I would need more information about it | | 76,6%(474) | 62,5%(40) | 1,961 (1,144 - 3,363) | 0,021 | 76,2%(128) | 74,3%(315) |  | 0,675 | 70,8%(170) | 70,8%(339) | 0,797 (0,642-0,990) | 0,046 |
| Economic resources | | 4,4%(21) | 5,9%(3) |  | 0,497 | 4,8%(6) | 3,5%(12) |  | 0,586 | 1,0%(2) | 1,0%(21) | 0,214 (0,57-0,809) | 0,002 |
| Do you know what the risks of MHT are? | | | | | |  |  |  |  |  | |  |  |
| It doesn't have any | | 23,0%(287) | 22,4%(32) |  | 0,917 | 26,6%(103) | 21,5%(184) | 1,323 (1,001 - 1,747) | 0,050 | 18,5%(101) | 18,5%(216) | 0,766 (0,642-0,990) | 0,002 |
| Breast cancer | | 35,4%(442) | 47,6%(68) | 0,605 (0,427 - 0,857) | 0,006 | 29,2%(113) | 41,4%(354) | 0,584 (0,451 - 0,755) | <0,001 | 46,4%(253) | 46,4%(257) | 1,487 (1,308-1,690) | <0,001 |
| Risk of thrombosis | | 32,0%(399) | 31,5%(45) |  | 0,925 | 29,5%(114) | 34,7%(297) |  | 0,069 | 43,1%(235) | 43,1%(208) | 1,6912 (1,420-1,829) | <0,001 |
| Osteoporosis | | 11,8%(147) | 13,3%(19) |  | 0,586 | 14,5%(56) | 9,5%(81) | 1,617 (1,123 - 2,327) | 0,011 | 7,2%(39) | 7,2%(127) | 0,566 (0,427-0,751) | <0,001 |
| Weight gain | | 42,0%(524) | 31,5%(45) | 1,576 (1,088 - 2,283) | 0,015 | 40,3%(156) | 40,1%(343) |  | 0,950 | 36,9%(201) | 36,9%(366) | 0,843 (0,735-0,967) | 0,013 |
| Cancer of the uterus | | 19,5%(243) | 21,0%(30) |  | 0,657 | 19,6%(76) | 18,7%(160) |  | 0,697 | 21,1%(115) | 21,1%(156) |  | 0,246 |
| Your sources of information on menopause are: | | | | | |  |  |  |  |  | |  |  |
| Friends | | 44,0%(874) | 39,7%(89) |  | 0,228 | 44,2%(265) | 43,3%(590) |  | 0,730 | 37,3%(307) | 37,3%(650) | 0,771 (0,688-0,863) | <0,001 |
| Magazines and press | | 26,2%(521) | 32,1%(72) |  | 0,067 | 24,8%(149) | 28,3%(386) |  | 0,111 | 31,4%(259) | 31,4%(330) | 1,253 (1,119-1,403) | <0,001 |
| Healthcare professionals | | 52,7%(1047) | 53,6%(120) |  | 0,832 | 53,8%323) | 54,2%(739) |  | 0,883 | 56,2%(463) | 56,2%(697) | 1,149 (1,030-1,281) | 0,012 |
| Family members | | 37,4%(742) | 25,4%(57) | 1,748 (1,276 - 2,392) | <0,001 | 38,0%(228) | 35,1%(478) |  | 0,221 | 33,7%(278) | 33,7%(519) |  | 0,058 |
| Television | | 16,3%(324) | 11,2%(25) | 1,552 (1,007 - 2,392) | 0,053 | 16,7%(100) | 15,7%(214) |  | 0,593 | 12,9%(106) | 12,9%(241) | 0,788 (0,666-0,933) | 0,004 |
| Internet | | 30,9%(614) | 33,0%(74) |  | 0,543 | 30,3%(182) | 32,1%(437) |  | 0,461 | 34,2%(282) | 34,2%(403) | 1,150 (1,028-1,286) | 0,016 |
| If you have marked healthcare professionals, specify | | | | | |  |  |  |  |  | |  |  |
| Gynecologist | | 66,0%(698) | 79,8%(95) | 0,491 (0,308 - 0,782) | 0,002 | 64,5%(209) | 69,3%(516) |  | 0,135 | 69,6%(320) | 69,6%(469) |  | 0,236 |
| Midwife | | 31,6%(327) | 7,0%(8) | 6,169 (2,972 - 12,804) | <0,001 | 25,2%(80) | 30,3%(220) |  | 0,102 | 27,2%(123) | 27,2%(209) |  | 0,276 |
| Nurse | | 23,2%(239) | 21,6%(25) |  | 0,729 | 22,7%(72) | 24,7%(179) |  | 0,529 | 29,6%(134) | 29,6%(128) | 1,403 (1,211-1,625) | <0,001 |
| Family doctor | | 36,8%(383) | 18,3%(21) | 2,609 (1,599 - 4,258) | <0,001 | 38,4%(123) | 32,7%(239) |  | 0,078 | 27,9%(126) | 27,9%(275) | 0,720 (0,610-0,850) | <0,001 |
| If you had any questions about menopause, who would you go to? | Primary health care | 31,9%(623) | 6,9%(16) | 6,353 (3,790 - 10,649) | <0,001 | 30,0%(182) | 29,1%(407) |  | 0,709 | 32,7%(196) | 32,7%(440) | 0,750 (0,658-0,855) | <0,001 |
|  | Specialty care | 68,1%(1330) | 93,1%(217) |  |  | 70,0%(425) | 70,9%(993) |  |  | 67,3%(632) | 67,3%(906) |  |  |
| Specify the reason: | | | | | |  |  |  |  |  | |  |  |
| Easy access | | 35,6%(747) | 24,4%(62) | 1,708 (1,266 - 2,306) | <0,001 | 36,5%(231) | 36,7%(529) |  | 0,961 | 37,0%(320) | 37,0%(487) |  | 0,055 |
| Trust | | 28,3%(595) | 39,8%(101) | 0,598 (0,458 - 0,783) | <0,001 | 34,6%(219) | 28,6%(412) | 1,324(1,084 - 1,616) | 0,007 | 27,7%(239) | 27,7%(452) |  | 0,135 |
| Knowledge in menopause | | 46,9%(986) | 58,7%(149) | 0,623 (0,479 - 0,811) | <0,001 | 47,2%(299) | 52,5%(757) | 0,811(0,673 - 0,978) | 0,032 | 57,6%(498) | 57,6%(632) | 1,119 (1,004-1,247) | <0,001 |
| Others | | 3,5%(73) | 1,6%(4) |  | 0,133 | 3,8%(24) | 3,5%(50) |  | 0,701 | 3,1%(27) | 3,1%(48) |  | 0,871 |

*n= Number of responses
