# Supplementary material for: Beliefs, knowledge and the impact of COVID19 on menopause therapies in Spanish women: COMEM-treatment study
Source: BMC Womens Health. 2020 Dec 28;20:277. doi: 10.1186/s12905-020-01151-x (PMC7768270; doi:10.1186/s12905-020-01151-x)
Supplement: Supplementary file 2 — Additional file 2. Table 4. Comparativeanalyses according to menopausal status, age and type of menopause. [file 12905_2020_1151_MOESM2_ESM.docx]

Table 4. Comparative analyses according to menopausal status, age and type of menopause

|  | |  | Menopausal status %(n*) | | | | Age | | | | Type of menopause %(n*) | | | |
| --- | --- | --- | --- | --- | --- | --- | --- | --- | --- | --- | --- | --- | --- | --- |
|  | |  | Postmenopause | Premenopause | OR (IC 95%) | p-value | Answer "yes"  Mean(SD**) | Answer "no"  Mean(SD**) | p-value | OR | Natural | Surgical | OR (IC 95%) | p-value |
| Are you interested in menopause? | | Yes | 83,1%(1085) | 89,2%(847) | 0,600 (0,467 - 0,771) | <0,001 | 51,9 (9,5) | 54,9 (12,4) | <0,001 | 1,031 (1,019 - 1,043) | 83,0%(899) | 84,5%(131) |  | 0,731 |
| What treatments do you know to treat the symptoms of menopause? | |  |  |  |  |  |  |  |  |  |  |  |  |  |
| No treatment exists | | | 4,1%(53) | 7,1%(65) | 0,557 (0,384 - 0,809) | 0,002 | 49,5 (12) | 52,7 (9,9) | <0,001 | 1,033 (1,013 - 1,053) | 4,2%(45) | 3,8%(6) |  | 1,000 |
| Vaginal lubricants | | | 57,6%(746) | 44,1%(403) | 1,72 (1,450 - 2,040) | <0,001 | 53,1 (9,3) | 51,9 (10,7) | <0,001 | 0,988 (0,980 - 0,996) | 57,1%(610) | 62,9%(710) |  | 0,170 |
| Hormonal treatment | | | 54,5%(706) | 59,3%(542) | 0,821 (0,692 -0,975) | 0,026 | 52,1 (9,5) | 53,1 (10,7) | 0,021 | 1,01 (1,002 - 1,018) | 54,7%(585) | 56,6%(90) |  | 0,670 |
| Antidepressants | | | 16,0%(207) | 15,0%(137) |  | 0,552 | 52,6 (10) | 52,5 (10) | 0,913 |  | 16,1%(172) | 14,5%(23) |  | 0,644 |
| Phytotherapy: soyisoflavones, red clover | | | 32,6%(423) | 26,3%(240) | 1,361 (1,128 - 1,642) | <0,001 | 53,1 (8,9) | 52,3 (10,4) | 0,067 |  | 33,7%(360) | 28,3%(45) |  | 0,206 |
| Acupuncture | | | 6,3%(82) | 7,3%(67) |  | 0,389 | 51,3 (10,2) | 52,6 (10) | 0,114 |  | 6,4%(68) | 6,3%(10) |  | 1,000 |
| Exercise: walking, yoga, etc. | | | 74,4%(964) | 65,2%(596) | 1,549 (1,288 - 1,863) | <0,001 | 52,9 (9,9) | 51,5 (10,3) | 0,003 | 0,986 (0,977 - 0,995) | 73,9%(790) | 77,4%(123) |  | 0,382 |
| Food rich in calcium and vitamin D | | | 69,0%(894) | 63,0%(576) | 1,305 ( 1,092 - 1,560) | 0,004 | 52,9 (10) | 51,8 (10) | 0,015 | 0,989 (0,980 - 0,998) | 67,6%(723) | 76,7%(122) | 0,634 (0,429 - 0,936) | 0,022 |
| Do you know what menopause hormone therapy (MHT) is? | | Yes | 53,0%(683) | 32,4%(304) | 2,358 (1,979 - 2,809) | <0,001 | 54,2 (9,5) | 50,9 (10,4) | <0,001 | 0,968 (0,960 - 0,976) | 52,9%(561) | 58,9%(96) |  | 0,177 |
| What are its indications? | | |  |  |  |  |  |  |  |  |  |  |  |  |
| Delay ageing | | | 16,6%(174) | 7,7%(55) | 2,386 (1,734 - 3,285) | <0,001 | 56,1 (10,6) | 52,1 (9,7) | <0,001 | 0,959 (0,946 - 0,973) | 17,3%(148) | 16,8%(23) |  | 1,000 |
| Improve hot flushes | | | 86,5%(907) | 87,4%(625) |  | 0,616 | 52,5 (9,8) | 53,1 (10,9) | 0,456 |  | 86,0%(737) | 85,4%(117) |  | 0,895 |
| Improve your sleep | | | 43,1%(452) | 39,4%(282) |  | 0,128 | 52,2 (9,1) | 52,9 (10,4) | 0,103 |  | 43,8%(375) | 43,1%(59) |  | 0,926 |
| Bones and joints improvement | | | 66,8%(701) | 63,2%(452) |  | 0,126 | 52,9 (10) | 52,1 (9,6) | 0,146 |  | 66,4%(569) | 72,3%(99) |  | 0,203 |
| Improve dyspareunia | | | 20,2%(212) | 14,4%(103) | 1,505 (1,163 - 1,947) | 0,002 | 53,7 (9,2) | 52,37 (10) | 0,026 | 0,986 (0,974 - 0,998) | 20,5%(176) | 20,4%(28) |  | 1,000 |
| Treating Depression | | | 15,9%(167) | 19,9%(142) | 0,764 (0,597 - 0,978) | 0,035 | 51,7 (10) | 52,8 (9,8) | 0,084 |  | 15,4%(132) | 18,2%(25) |  | 0,380 |
| Would you use MHT if your gynecologist advised you | | Yes | 70,0%(883) | 83,3%(764) | 0,467 (0,378 - 0,576) | <0,001 | 51,5 (10,1) | 55,2 (9,5) | <0,001 | 1,038 (1,027 - 1,048) | 69,3%(721) | 72,6%(114) |  | 0,456 |
| If the answer is no, specify the reasons: | |  |  |  |  |  |  |  |  |  |  |  |  |  |
| Fear and distrust | | | 33,2%(124) | 24,5%(37) |  | 0,060 | 56,3 (9,7) | 54,9 (9,3) | 0,106 |  | 31,6%(100) | 45,0%(18) |  | 0,109 |
| No need to treat it | | | 20,7%(78) | 6,7%(10) | 3,638 (1,828 - 7,242) | <0,001 | 57,7 (9,6) | 54,9 (9,35) | 0,013 | 0,969 (0,945 - 0,993) | 21,0%(67) | 20,0%(8) |  | 1,000 |
| I would need more information about it | | | 68,7%(320) | 89,0%(187) | 0,270 (0,168 - 0,434) | <0,001 | 54 (9,7) | 57,8 (9,4) | <0,001 | 1,042 (1,023 - 1,061) | 69,4%(275) | 64,6%(31) |  | 0,511 |
| Economic resources | | | 4,3%(16) | 4,7%(7) |  | 0,816 | 54,7 (12,2) | 55,3 (9,4) | 0,761 |  | 4,1%(13) | 4,9%(2) |  | 0,686 |
| Do you know what the risks of MHT are? | | |  |  |  |  |  |  |  |  |  |  |  |  |
| It doesn't have any | | | 17,6%(147) | 31,1%(169) | 0,475 (0,369 - 0,613) | <0,001 | 51 (10,9) | 53,4 (10) | <0,001 | 1,024 (1.011 - 1,037) | 18,3%(125) | 16,1%(18) |  | 0,690 |
| Breast cancer | | | 43,1%(359) | 27,0%(147) | 1,619 (2,584 - 0,000) | <0,001 | 54 (9,5) | 52,2 (10,6) | 0,002 | 0,983 (0,973 - 0,994) | 41,7%(285) | 50,0%(56) |  | 0,101 |
| Risk of thrombosis | | | 33,1%(276) | 30,0%(163) |  | 0,237 | 52,5 (10) | 53,1 (10,4) | 0,356 |  | 33,8%(231) | 25,9%(29) |  | 0,104 |
| Osteoporosis | | | 13,8%(115) | 9,4%(51) | 1,548 (1,092 - 2,195) | 0,014 | 56,1 (11,1) | 52,4 (10) | <0,001 | 0,965 (0,950 - 0,981) | 13,5%(92) | 15,2%(17) |  | 0,656 |
| Weight gain | | | 40,2%(335) | 41,5%(226) |  | 0,654 | 52,9 (10,3) | 52,8 (10,3) | 0,888 |  | 39,8%(272) | 43,8%(49) |  | 0,467 |
| Cancer of the uterus | | | 22,9%(191) | 14,5%(79) | 1,751 (1,313 - 2,336) | <0,001 | 53,3 (9,7) | 52,8 (10,4) | 0,507 |  | 22,7%(155) | 25,0%(28) |  | 0,628 |
| Your sources of information on menopause are: | | |  |  |  |  |  |  |  |  |  |  |  |  |
| Friends | | | 41,1%(522) | 47,0%(434) | 0,786 (0,663 - 0,933) | 0,006 | 52 (9,7) | 52,4 (10,4) | 0,241 |  | 41,9%(440) | 38,9%(61) |  | 0,488 |
| Magazines and press | | | 29,4%(374) | 23,2%(214) | 1,383 (1,138 - 1,680) | 0,001 | 53,4 (9,3) | 52 (10,3) | 0,003 |  | 28,9%(303) | 32,5%(51) |  | 0,349 |
| Healthcare professionals | | | 61,7%(783) | 40,7%(376) | 2,339 (1,967 - 2,782) | <0,001 | 53,7 (9,6) | 50,8 (10,4) | <0,001 | 0,971 (0,963 - 0,979) | 61,5%(645) | 64,3%(101) |  | 0,538 |
| Family members | | | 29,0%(368) | 46,5%(429) | 0,470 (0,393 - 0,561) | <0,001 | 50,2 (10,3) | 53,5 (9,8) | <0,001 | 1,035 (1,026 - 1,044) | 28,2%(296) | 33,1%(52) |  | 0,220 |
| Television | | | 15,9%(202) | 15,7%(145) |  | 0,953 | 52,1 (10,3) | 52,4 (10) | 0,600 |  | 15,3%(160) | 14,6%(23) |  | 0,906 |
| Internet | | | 28,3%(360) | 34,8%(321) | 0,742 (0,618 - 0,890) | 0,001 | 50,1 (8,4) | 53,3 (10,6) | <0,001 | 1,033 (1,024- 1,043) | 27,6%(289) | 31,8%(50) |  | 0,295 |
| If you have marked healthcare professionals, specify | | |  |  |  |  |  |  |  |  |  |  |  |  |
| Gynecologist | | | 67,6%(535) | 66,9%(253) |  | 0,841 | 53,7 (9,4) | 53,8 (9,7) | 0,831 |  | 66,4%(434) | 77,8%(77) | 0,564 (0,342-0,930) | 0,028 |
| Midwife | | | 26,5%(205) | 34,6%(128) | 0,681 (0,521 - 0,890) | 0,005 | 52 (9,2) | 54,4 (9,5) | <0,001 | 1,027 (1,013 - 1,042) | 28,4%(181) | 10,1%(10) | 3,525 (1,793-9,930) | <0,001 |
| Nurse | | | 21,3%(164) | 26,7%(98) | 0,743 (0,557 - 0,991) | 0,050 | 52,3 (9,8) | 54,1 (9,4) | 0,007 | 1,02 (1,005 - 1,035) | 21,1%(134) | 22,2%(22) |  | 0,793 |
| Family doctor | | | 36,6%(285) | 32,2%(119) |  | 0,165 | 54,7 (9,4) | 53,2 (9,5) | 0,014 | 0,984 (0,971 - 0,997) | 37,5%(241) | 37,4%(37) |  | 1,000 |
| If you had any questions about menopause, who would you go to? | Primary health care | | 31,9%(623) | 6,9%(16) | 6,353 (3,790 - 10,649) | <0,001 | 53 (10) |  | 0,045 | 0,991 (0,982 - 1,000) | 31,5%(326) | 25,8%(41) |  | 0,166 |
|  | | Specialty care | 68,1%(586) | 93,1%(93) |  |  | 52,1 (10,2) |  |  |  | 68,5%(709) | 74,2%(118) |  |  |
| Specify the reason: | | |  |  |  |  |  |  |  |  |  |  |  |  |
| Easy access | | | 32,7%(445) | 36,6%(356) |  | 0,052 | 51,9 (9,9) | 52,8 (10,2) | 0,038 | 1,009 (1,001 - 1,018) | 33,3%(375) | 29,2%(49) |  | 0,292 |
| Trust | | | 31,7%(432) | 26,6%(259) | 1,281 (1,067 - 1,537) | 0,009 | 53,4 (10,3) | 52,1 (10) | 0,005 | 0,988 (0,979 - 0,996) | 31,5%(354) | 37,5%(63) |  | 0,132 |
| Knowledge in menopause | | | 46,7%(636) | 50,5%(491) |  | 0,078 | 52 (10,1) | 53 (10,2) | 0,021 | 1,009 (1,001 - 1,018) | 45,8%(515) | 51,2%(86) |  | 0,214 |
| Others | | | 2,6%(36) | 4,2%(41) | 0,617 (0,391 - 0,973) | 0,045 | 49,4 (9,6) | 52,6 (10,1) | 0,005 | 1,034 (1.010 - 1,058) | 2,6%(29) | 3,6%(6) |  | 0,444 |

*n= Number of responses

SD** standard desviation
